# Mental Imagery Enhances Pain Reduction and Visual Processing in Knee Osteoarthritis Patients: A Comparative Study

**DOI:** 10.1155/prm/5576698

**Published:** 2025-07-22

**Authors:** Gülsüm Akdeniz, Kıvanç Tığlı, Nur Efşan Akıncı, Halil Kul, Melih Çamcı, Harun Demirci, Sevgi İkbali Afşar

**Affiliations:** ^1^Faculty of Medicine, Department of Neuroscience, Department of Biophysics, Ankara Yildirim Beyazit University, Ankara, Türkiye; ^2^Faculty of Medicine, Department of Neuroscience, Ankara Yildirim Beyazit University, Ankara, Türkiye; ^3^Faculty of Medicine, Department of Physical Therapy and Rehabilitation, Başkent University, Ankara, Türkiye; ^4^Faculty of Medicine, Department of Neuroscience, Department of Neurosurgery, Ankara Yildirim Beyazit University, Ankara, Türkiye; ^5^Faculty of Medicine, Department of Neuroscience, Department of Emergency Medicine, Ankara Yildirim Beyazit University, Ankara, Türkiye

## Abstract

**Objective:** Mental imagery involves forming internal sensory representations, while osteoarthritis is a degenerative joint disease characterized by cartilage loss. This study explores how mental imagery can modulate pain perception and enhance visual processing in individuals with knee osteoarthritis.

**Methods:** Forty-eight participants were randomly assigned to a mental imagery group or a treatment group. The treatment group received conventional physiotherapy interventions, including ultrasound, transcutaneous electrical nerve stimulation, hot pack application, and isometric knee exercises, while the mental imagery group mentally imagined the same treatments. Both groups underwent interventions for 10 days, with assessments before and after. Pain intensity was measured using the visual analog scale (VAS), and visual processing was assessed through the digital pareidolia test.

**Results:** Both groups exhibited significant reductions in VAS scores, with the mental imagery group demonstrating a more substantial decrease. Notably, the mental imagery group had faster reaction times to face pareidolia images, indicating improved visual processing. In contrast, the treatment group's reaction times to face pareidolia images remained unchanged.

**Conclusion:** These findings highlight that mental imagery could serve as an alternative approach to pain management and cognitive enhancement, potentially influencing top-down mechanisms in facial pattern recognition. This highlights the potential for mental imagery to be integrated into therapeutic strategies for pain-related conditions, promoting personalized, innovative treatments.

## 1. Introduction

Mental imagery, a cognitive process involving the formation and manipulation of internal representations across sensory modalities [1–3], plays a critical role in diverse cognitive functions, including memory, learning, spatial reasoning, and its intricate connection to emotions and decision-making [3, 4]. While the vividness and controllability of these internal depictions vary interindividually and can be influenced by expertise [3], mental imagery, distinct from imagination as a perceptual representation [1], has significant implications in fields such as clinical neuroscience and social cognition [5–7]. Building upon the understanding of visual processing inherent in mental imagery is the phenomenon of pareidolia, wherein individuals perceive meaningful patterns in random stimuli through the interplay of bottom-up and top-down cognitive mechanisms [8, 9]. A prominent manifestation of this is face pareidolia, the illusory perception of faces, underscoring the salience of facial processing in social interactions. The digital pareidolia test (PT) provides a methodology for assessing both face and face pareidolia, yielding valuable data on related cognitive and emotional processing [10]; however, its clinical application in conjunction with imaginary treatment methods remains an unexplored area of research.

Osteoarthritis (OA) is a prevalent degenerative joint disease characterized by the breakdown of cartilage, especially in load-bearing joints, leading to pain and functional impairment, and is most commonly observed in hip and knee joints [11]. OA is influenced by both intrinsic factors, such as age [12], gender [13], and genetics [14], and extrinsic factors, including obesity [15] and lifestyle choices [16]. While intrinsic factors are largely nonmodifiable, addressing extrinsic factors presents opportunities for intervention, OA prevention, and management. The symptoms of OA, particularly in the knee, are significant contributors to a reduced quality of life and increased healthcare burden. The primary symptoms include pain [17], stiffness [18], and limited joint movement [19], which can severely impact daily activities and work capacity. Knee OA represents a prevalent chronic joint disorder distinguished by elevated rates of morbidity and disability, predominantly affecting the knee joint, which experiences the highest mechanical load, resulting in irreversible degeneration of the articular cartilage, synovium, and surrounding structures [20].

Building upon the established utility of mental imagery in cognitive and emotional processes, and considering its potential in clinical interventions, traditional physiotherapy practices for managing knee OA, which typically involve manual therapy, exercises, and patient education to reduce pain and improve function [21, 22], are now being complemented by innovative imagery-based interventions [23–25]. While imagination can serve as a distractive and relaxing cognitive strategy [26], motor imagery, a specific form of mental imagery involving the cognitive simulation of movement without physical execution, has demonstrated efficacy in facilitating motor learning and recovery under various conditions [27]. Evidence suggests that incorporating imagery into physiotherapeutic protocols can lead to significant improvements in pain reduction, functional mobility, and muscle strength, particularly in postsurgical rehabilitation following total knee arthroplasty [24, 25, 27]. Neuroimaging studies, such as functional magnetic resonance imaging, have further elucidated the neural underpinnings of this phenomenon, revealing overlapping brain activation patterns during imagery and actual motor execution [28].

We aim to explore the capacity for sensory perceptive activity, specifically focusing on the role of mental imagery as a mechanism for modulating pain perception and facilitating visual processing inherent in mental imagery.

## 2. Methods

The study was conducted on patients at the Physical Therapy and Rehabilitation Clinic of Başkent University Ankara Hospital. The sample size of the study was calculated using G^∗^Power (Version 3.0.10; Franz Foul, Universitat Kiel, Germany). As a result of the power analysis, the sample size was 24 in each group and 48 individuals in total, with a large effect size (*α* = 0.05 and 85% power). Ethical approval was received from the Başkent University Medical and Health Sciences Research Board and Ethics Committee (Project no.: KA23/441) and supported by the Başkent University Research Fund.

### 2.1. Participants

**Figure 1:**
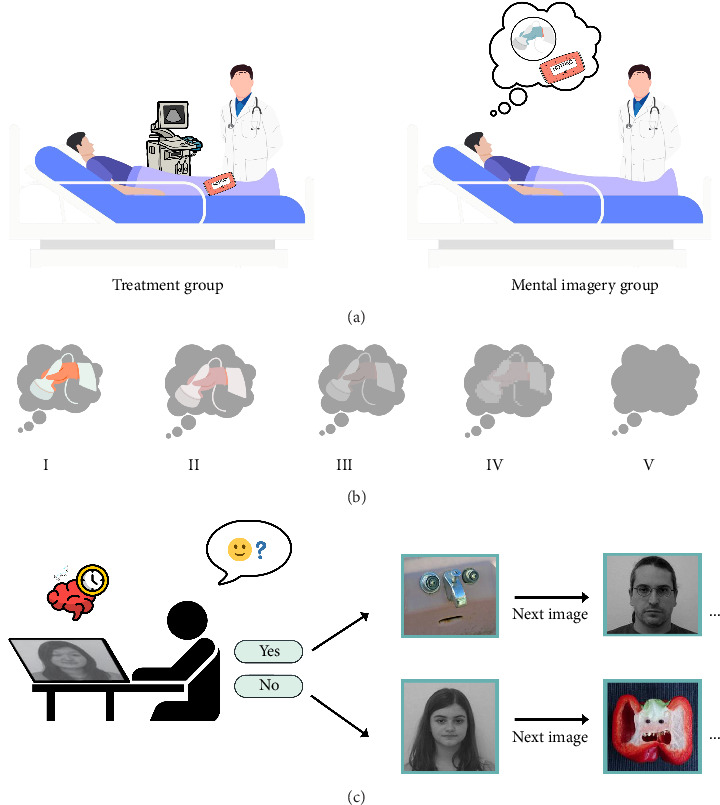
(a) Patients received ultrasound, TENS, hot pack application, and isometric knee exercises in the treatment group while patients followed a guided imagery protocol, mentally visualizing treatment steps with eyes closed under verbal guidance in the mental imagery group. (b) Diagram depicting the spectrum of mental imagery ranging from fully formed imagery to complete absence thereof (I–IV) based on individuals' self-regulatory capacities derived from their imaginative experiences. (c) Participants indicated whether they perceived a face in each pareidolia and face images and responses with reaction times were digitally recorded.

Classical physical therapy applications and treatment imagination were applied to 48 patients (24 females and 24 males)who participated in the study voluntarily. The study comprised patients who were diagnosed with knee OA, aged 50–65 years, and did not have a neurological or psychiatric disease. Patients who were not volunteering to participate, had orthopedic surgery on the knee, and had a psychiatric diagnosis were excluded from the study. The study participants were informed about the research and filled out an informed consent form. The patients were randomly allocated into two groups: the mental imagery group and the treatment group (Figure 1(a)). Randomization was performed using a simple random allocation method without stratification for sex or age. Both the treatment group, receiving ten sessions of traditional physiotherapy 5 days per week, and the mental imagery group, receiving the same frequency of mental imagery intervention, underwent comprehensive assessments twice, pre- and postintervention.

### 2.2. Measures

#### 2.2.1. The Visual Analog Scale (VAS)

**Figure 2:**
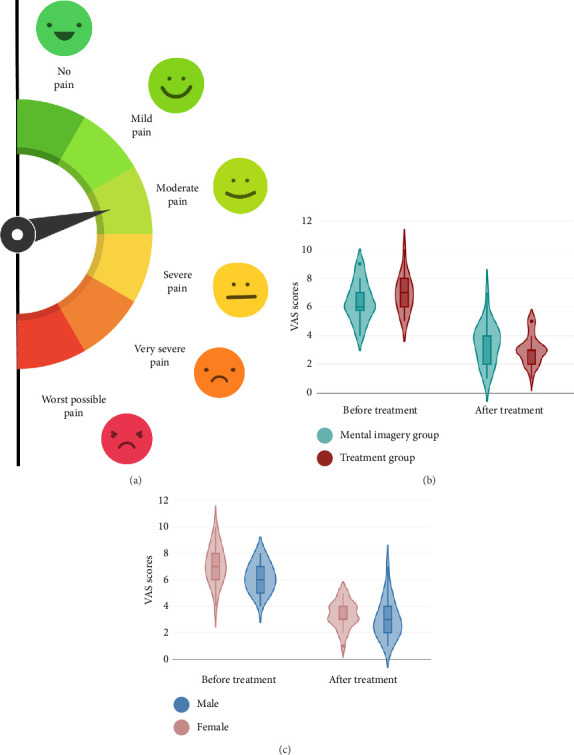
(a) Digital VAS scores from no pain to worst possible pain. (b) Violin plots of the changes in the visual analog scale (VAS) at pre- and postcondition in the treatment group (VAS_pre: 6.92 ± 1.24; VAS_post: 3.00 ± 1.02) and the mental imagery group (VAS_pre: 6.38 ± 1.37; VAS_post: 3.42 ± 1.41). (c) Violin plots of the changes in the visual analog scale (VAS) at pre- and postcondition in both groups regarding the gender of the participants.

VAS was used to measure the pain intensity of the patients from no pain to worst possible pain. The scale is 10 cm long and has different descriptors at both ends on a horizontal line (Figure 2(a)). The patients marked the point representing the pain intensity on the line, and the pain levels of the patients were recorded before and after the 10 sessions of treatment and imagery interventions.

#### 2.2.2. Digital PT

Patients were presented with a randomized sequence of 30 images, comprising 10 face images, 10 face pareidolia images, and 10 scrambled images (nontarget images) which were selected from the previous study [10]. All images were converted to greyscale with the same resolution, position, luminance, and contrast. The visual stimuli were standardized to a dimension of 384 × 384 pixels. All images were standardized in terms of size, tone, and light intensity. These images were presented in a digital format through the Qualtrics CoreXM platform (Qualtrics, Seattle, Washington, USA). Participants were instructed to indicate whether they perceived a face within the image; if their response is “No,” the subsequent image would be presented, while their responses and corresponding reaction times were recorded digitally (Figure 1(c)).

### 2.3. Interventions

#### 2.3.1. Traditional Physiotherapy Application

Patients in this treatment group were treated with ultrasound, transcutaneous electrical nerve stimulation (TENS), hot pack application, and isometric knee exercises with lasting an average of 45 min in total.

• Ultrasound: The ultrasound gel was applied to the transducer and then moved in a circle over the patient's affected area. The ultrasound modality utilized was continuous, operating at a frequency of 1 MHz, with an intensity range of 1–1.5 W/cm^2^ for a duration of 10 min.
• TENS: The designated area for treatment was adequately exposed, ensuring the rest of the body remained covered. Silicone rubber electrodes were affixed to the skin utilizing adhesive tape. The dermatomal placement technique was employed. One electrode was strategically positioned at the corresponding spinal nerve root level, while the other was placed at the distal end of the dermatome. The conventional TENS was utilized, characterized by a pulse duration of 50 microseconds and a frequency of 30–40 Hz. The application was conducted in continuous mode and lasted for a duration of 20 min.
• Hot Pack Application: A superficial heating agent (hot pack) was applied to the knee region of the patients for 20 min.
• Isometric Knee Exercises: Exercise commenced with isometric contractions targeting the knee flexors, lateral flexors, rotators, and extensors. These contractions were sustained for a duration of 5 s. The participants were instructed to execute 10 repetitions in each direction. The exercises lasted for a duration of 10 min.

#### 2.3.2. Mental Imagination Application

Patients in this mental imagery group underwent a guided mental imagery protocol, in which they imagined the real treatment processes step by step while keeping their eyes closed by the practitioner with verbal commands. An illustrative diagram depicting the spectrum of mental imagery, ranging from fully formed imagery to complete absence thereof (I–IV), based on individuals' self-regulatory capacities derived from their imaginative experiences, is presented in Figure 1(b). Active engagement duration in each guided mental imagery session was recorded using standardized time logs by the practitioner, confirming that all participants completed the full 45-min session. Participants in the mental imagery group received only imagery-based rehabilitation without any conventional physiotherapy during the intervention period. Mental simulation was conducted in a quiet room with minimal distractions, and participants were provided with verbal guidance to ensure accurate and detailed visualization of each treatment component. This process was structured as follows:

• Mental Simulation of Ultrasound: Participants were instructed to imagine the application of ultrasound gel onto their affected area, followed by the movement of a transducer in circular motions over the same region. They were guided to visualize the sensation of warmth and the penetration of ultrasonic waves into the tissues. The imagined ultrasound parameters were described as continuous mode with a frequency of 1 MHz and an intensity of 1–1.5 W/cm^2^. The guided imagery session for this phase lasted for 10 min.
• Mental Simulation of TENS: Participants were asked to imagine the exposure of the designated treatment area, while the rest of their body remained covered. They were guided to visualize the placement of silicone rubber electrodes on their skin, one at the corresponding spinal nerve root level and the other at the distal end of the dermatome. Participants were encouraged to imagine the sensation of mild electrical stimulation, as typically felt during TENS. They were reminded of the technical details, including a pulse duration of 50 ms, a frequency of 30–40 Hz, and continuous mode application. The mental simulation for this phase lasted for 20 min.
• Mental Simulation of Hot Pack Application: Participants were guided to visualize the placement of a warm hot pack on their knee region. They were encouraged to mentally experience the gradual sensation of warmth spreading through their tissues, similar to a real-life application. This imagery session lasted for 20 min.
• Mental Simulation of Isometric Knee Exercises: Participants were instructed to mentally perform isometric contractions of the knee flexors, lateral flexors, rotators, and extensors. They were guided to imagine engaging each muscle group sequentially, holding each contraction for 5 s. The instructions included mentally counting 10 repetitions in each direction. The duration of this mental simulation was 10 min.

Throughout the session, participants were encouraged to maintain focused attention on their mental imagery and to vividly engage their sensory and motor representations associated with the treatment procedures. The guided imagery was conducted by a trained practitioner to ensure consistency and adherence to the protocol. Any instances of distraction or difficulty in visualization were addressed with verbal prompts to enhance engagement and accuracy.

### 2.4. Statistical Analysis

The obtained data were evaluated using the Statistical Package for the Social Sciences (SPSS) Version 22.0. Comparisons were made between the groups. The statistical significance level was accepted as *p* < 0.05. The normality of data distribution was assessed using the Shapiro–Wilk test, which confirmed that all variables met the assumption of normality (*p* > 0.05), thereby justifying the use of parametric tests. For the data obtained in the study, independent samples *t*-test and paired sample *t*-test were used for VAS and digital PT values.

## 3. Results

A total of 48 participants (24 females and 24 males) were included in the study, with the mental imagery group consisting of 13 males and 11 females (mean age = 58.25) and the treatment group comprising 11 males and 13 females (mean age = 59.79). Demographic information of the participants included in the study is shown in Table 1. An independent samples *t*-test was conducted to assess baseline group equivalence in pain severity, and no significant difference was found in pretreatment VAS scores between the mental imagery group (6.41 ± 1.37) and the treatment group (6.85 ± 1.29) (*t* (46) = 1.14, *p*=0.261).

Repeated-measures ANOVA revealed a significant main effect of time on VAS scores (*p* < 0.001, *η*_*p*_^2^ = 0.880), but no significant main effect of group (*p*=0.242) or time × group interaction (*p*=0.367) was observed (Table 2). Although the time × group interaction did not reach statistical significance (*p*=0.057), paired samples *t*-tests showed significant reductions in VAS scores for both the treatment (6.84 to 3.07) and mental imagery (6.40 to 3.36) groups (*p* < 0.05; Figure 2(b)). Notably, female participants in both groups had higher pretreatment VAS scores compared to male participants (Figure 2(c)).

Regarding reaction times to face stimuli, paired samples *t*-tests showed a significant reduction in the mental imagery group (1.04 ± 0.30 to 0.91 ± 0.13; *p*=0.030), whereas the treatment group showed a nonsignificant decrease from 1.10 to 1.05 s (*p*=0.329, Table 3). However, repeated-measures ANOVA did not reveal a significant time × group interaction for this variable (*p*=0.185), and between-group differences at posttreatment were not statistically significant (*p*=0.187; Table 2).

In contrast, for face pareidolia stimuli, ANOVA showed a significant main effect of time (*p*=0.025, *η*_*p*_^2^ = 0.105), but no significant effects of group or interaction (*p* > 0.05). As a result of the paired sample *t*-test, there was no significant difference in pre- and posttreatment reaction times to face stimuli in the mental imagery and treatment groups (*p* > 0.05). However, posttreatment comparison between the groups revealed that the treatment group had significantly slower reaction times in face pareidolia compared to the mental imagery group (*t* = 2.13, *p* < 0.05; Table 4).

## 4. Discussion

Our research is the first comparative analysis of treatment and mental imagery intervention for modulating pain perception and enhancing visual processing in patients with knee OA, using a novel digital PT evaluation. The results of this study highlight that assessments of pain and visual perception revealed statistically significant improvements in both pain intensity and visual processing acuity following a ten-day intervention period in both the treatment group and the mental imagery group. Notably, our striking finding underscores the substantive role of mental imagery, as evidenced by the mental imagery group exhibiting a statistically significant reduction in pain scores and a more pronounced enhancement in visual processing efficiency when contrasted with the treatment group. We interpret our results that mental imagery exerts a significant influence on our perception of reality by shaping the interpretation of sensory information. It enables individuals to self-regulate their perceptions, particularly in the context of pain management. Our findings indicate that sensory-perceptive activity is integral to shaping an individual's perception of reality, facilitating adaptation based on personal experiences. This underscores the significance of perception in processing both external and internal stimuli. We emphasize that mental imagery can serve as an effective method for pain management, as it influences neuronal processes that generate genuine sensory experiences. This mental imagery method could pave the way for personalized innovative therapy strategies that cater to individual cognitive profiles, ultimately fostering a more holistic approach to the management of pain-related disorders.

Our findings revealed significant reductions in pain intensity in both the mental imagery and treatment groups following the interventions, demonstrating that both applications positively affected patients' pain status and were effective in managing pain in patients with knee OA. A study by Aydın and Doğan [29] investigated the impact of guided imagery on managing postoperative pain in 30 patients who underwent lower extremity surgical operations. Patients were randomly assigned to either a guided imagery intervention group which mentally visualize to create calming and positive images in the mind, or a control group. Following the intervention period, pain levels were assessed using the VAS and their results demonstrated a statistically significant reduction in pain for the intervention group compared to the control group. They suggested that incorporating guided imagery into postoperative care can be beneficial for pain management and has positive effects on emotional well-being during recovery. Another study by Biéchy et al. [30] investigated the effectiveness of an experimental recovery protocol combining deep breathing and mental imagery on cardiovascular recovery in 40 firefighters after intense physical activity, comparing it to a control recovery protocol. The experimental recovery protocol showed significant improvements in heart rate recovery and parasympathetic reactivation compared to the control recovery protocol. They suggest that incorporating deep breathing and mental imagery can enhance physical and potentially psychological recovery in firefighters, advocating for their inclusion in postactivity recovery routines. Considering these findings, our results further support the growing body of evidence highlighting the efficacy of mental imagery interventions in pain reduction. We suggest that the mental imagery approach can improve pain management, emotional well-being, motivation in patients, and overall recovery experiences. Moreover, healthcare providers may consider incorporating mental imagery into pain management and recovery protocols across various patient populations as a potential alternative approach in rehabilitation programs. Visual mental imagery may be used to heal from illness because it engages the mind in a way that can influence physical processes, fostering a sense of control and empowerment in patients. By imagining positive scenarios related to their health, patients may experience reduced stress levels, which can enhance immune function and promote overall well-being. Our suggested intervention not only aids in recovery but may also encourage a proactive approach to health, allowing patients to actively participate in their healing journey through focused visualization and positive thinking.

Our surprising findings reveal a notable acceleration in reaction times to face pareidolia images within the mental imagery group following the imaginary intervention, a change not mirrored by a statistically significant reduction in the treatment group receiving actual physiotherapy. While reaction times to genuine face stimuli remained comparable between pre- and postintervention within both groups, a direct posttreatment comparison highlighted a significant advantage in the mental imagery group, demonstrating faster processing of face pareidolia compared to the treatment group. A study by Lakshminarayanan et al. [31] aimed to determine whether tactile imagery (TI) training, which involves mentally simulating a tactile sensation, could effectively improve reaction time in healthy participants. Participants' reaction times to vibratory stimuli were measured before and after a TI training session. An experimental group underwent TI training, while a control group did not receive any training, and they compared the changes in reaction time pre- and posttraining within the experimental group and against the control group. They found that TI training led to a significant improvement in reaction time, with a reduction of approximately 25% after training. They suggest that mental training techniques like TI may have valuable applications in improving sensorimotor skills. The occipitotemporal regions, which include the primary visual cortex, play a key role in how we perceive visual information and are strongly linked to bottom-up processing [32]. In contrast, the frontal cortex is where reasoning occurs and is closely associated with top-down processing [33, 34]. When we encounter illusory face stimuli, those featuring elements similar to real faces, like eyes and mouths, we engage in top-down processing. This allows us to draw on our prior knowledge of actual faces, leading us to interpret these illusions as real faces [8]. Considering our findings alongside the existing literature, it becomes evident that mental imagery interventions can selectively enhance cognitive processing, particularly in the context of ambiguous or illusory visual stimuli such as face pareidolia. This suggests that mental imagery may prime top-down mechanisms involved in facial pattern recognition, thereby facilitating faster perceptual processing. We interpret our result that mental imagery may act as a cognitive enhancer by modulating top-down pathways involved in the interpretation of facial patterns, thereby facilitating faster perceptual processing. Hence, imagery-based interventions may offer particular advantages in contexts where patients experience pain, serving as a viable option for addressing the perceptual dimension of pain and facilitating enhanced comfort in disease management. Our findings show that visual mental imagery has been effectively utilized in health settings to aid rehabilitation and pain management and enhance overall well-being. Furthermore, techniques such as guided imagery and visualization are employed to help patients reduce their anxiety, improve their coping strategies during medical procedures, and promote healing by mentally envisioning positive outcomes and recovery processes.

A limitation of the study is that it assessed outcomes only immediately after the intervention period; long-term follow-up would be valuable to determine whether the effects are sustained over time. Moreover, the study focused on patients aged 50–65 years with knee OA, limiting the generalizability of the findings to other age groups or clinical populations. Physiological validation of the mental imagery intervention such as skin temperature or conductance changes was not performed, which could have provided objective evidence of autonomic engagement, and EMG recordings were not collected during motor imagery tasks to confirm the absence of actual muscle contractions. Finally, future studies are encouraged to include standardized tools to evaluate individual differences in imagery vividness and engagement.

## 5. Conclusion

A multidisciplinary framework that embraces both physical exercise and neuropsychological support can pave the way for future research into integrative therapy models, ultimately benefiting those affected by this degenerative joint disease. Thus, exploring the synergistic effects of these treatment modalities could lead to improved patient outcomes and lower burden on healthcare systems. Additionally, the incorporation of techniques such as visualization and mental imagery can empower patients to feel more in control of their prognosis, promoting a proactive attitude toward managing their OA symptoms. Future research should consider investigating the long-term effects of mental imagery on pain perception and cognitive processing, as well as exploring its application in various patient populations and conditions. Our findings warrant further exploration of the neural mechanisms underlying these effects and the potential for integrating mental imagery with other therapeutic strategies to optimize patient outcomes in clinical settings.

## Figures and Tables

**Table 1:** Demographic information of patients.

Variables	Experiment group (*n* = 24)	Treatment group (*n* = 24)	Total (*n* = 48)
*Gender*, *n* (%)			
Male	13 (54.2%)	11 (45.8%)	24 (50%)
Female	11 (45.8%)	13 (54.2%)	24 (50%)
Age (years), avg./sd.	58.25 ± 4.74	59.79 ± 3.92	59.02 ± 4.37

*Education level, n (%)*			
Primary school	0 (0.0%)	2 (8.3%)	2 (4.2%)
Secondary school	2 (8.3%)	3 (12.5%)	5 (10.4%)
High school	7 (29.2%)	10 (41.7%)	17 (35.4%)
University degree	15 (62.5%)	9 (37.5%)	24 (50.0%)

*Etiology grade, n (%)*			
First grade	13 (54.2%)	10 (41.7%)	23 (47.9%)
Second grade	9 (37.5%)	12 (50.0%)	21 (43.8%)
Third grade	2 (8.3%)	2 (8.3%)	4 (8.3%)
Disease duration (years), avg./sd.	5.96 ± 3.76	6.12 ± 2.88	6.04 ± 3.31
Weight (kg), avg./sd.	77.54 ± 10.07	76.96 ± 9.91	77.25 ± 9.89
Height (cm), avg./sd.	1.71 ± 0.08	1.70 ± 0.07	1.70 ± 0.07

*Note:* avg.; average, sd.; standard deviation, kg = kilogram; cm = centimeter.

**Table 2:** Repeated-measures ANOVA results for behavioral outcomes.

Measure		*F* (1, 46)	*p*	*η* ^2^ *p*
VAS score	Time (pre-int vs. post-int)	336.86	0.001^∗^	0.880
	Group (imagery vs. treatment)	0.05	0.817	0.001
	Time × group	3.80	0.057	0.076

Face images reaction time	Time (pre-int vs. post-int)	0.04	0.831	0.001
	Group (imagery vs. treatment)	2.48	0.122	0.051
	Time × group	1.81	0.185	0.038

Face pareidolia images reaction time	Time (pre-int vs. post-int)	5.38	0.025^∗^	0.105
	Group (imagery vs. treatment)	1.40	0.242	0.030
	Time × group	0.83	0.367	0.018

*Note:* Time = preintervention vs. postintervention; group = imagery vs. treatment; *η*^2^*p* = partial eta-squared.

^∗^ *p* < 0.05 indicates significance.

**Table 3:** Comparison of face and face pareidolia reaction times within the groups before and after treatment.

	Before treatment Mean ± SD	After treatment Mean ± SD	*t* (df)	*p*	Cohen's *d*
*Face*					
Mental imagery group	1.04 ± 0.30	0.91 ± 0.13	*t* (21) = 2.32	0.030^∗^	0.495
Treatment group	1.10 ± 0.49	1.05 ± 0.31	*t* (25) = 0.99	0.329	0.195

*Face pareidolia*					
Mental imagery group	1.18 ± 0.32	0.11 ± 0.29	*t* (21) = 0.92	0.370	0.195
Treatment group	1.25 ± 0.46	1.34 ± 0.42	*t* (25) = −1.03	0.312	−0.202

*Note:* Paired sample *t* test.

^∗^ *p* < 0.05.

**Table 4:** Comparison of face pareidolia reaction times between groups before and after treatment.

	Mental imagery group	Treatment group	*t* (df)	*p*	Cohen's *D*
	Mean ± SD	Mean ± SD			
Before treatment	1.04 ± 0.30	1.10 ± 0.49	*t* (46) = 0.56	0.575	−0.406
After treatment	0.90 ± 0.13	1.05 ± 0.32	*t* (46) = 2.13	0.040^∗^	−0.002

*Note:* Independent samples *t* test.

^∗^ *p* < 0.05.

## Data Availability

The data that support the findings of this study are available from the corresponding author upon reasonable request.
